# Terahertz Driven Reversible Topological Phase Transition of Monolayer Transition Metal Dichalcogenides

**DOI:** 10.1002/advs.202003832

**Published:** 2021-04-02

**Authors:** Jian Zhou, Haowei Xu, Yongliang Shi, Ju Li

**Affiliations:** ^1^ Center for Alloy Innovation and Design Center for Advancing Materials Performance from the Nanoscale State Key Laboratory for Mechanical Behavior of Materials Xi'an Jiaotong University Xi'an 710049 China; ^2^ Department of Nuclear Science and Engineering Massachusetts Institute of Technology Cambridge MA 02139 USA; ^3^ Center for Spintronics and Quantum Systems State Key Laboratory for Mechanical Behavior of Materials Xi'an Jiaotong University Xi'an 710049 China; ^4^ Department of Materials Science and Engineering Massachusetts Institute of Technology Cambridge MA 02139 USA

**Keywords:** density functional theory, optical response, terahertz optics, topological phase transition, transition metal dichalcogenide

## Abstract

This paper shows how terahertz light can drive ultrafast topological phase transitions in monolayer transition metal dichalcogenides (TMDs). The phase transition is induced by the light interaction with both electron and phonon subsystems in the material. The mechanism of such a phase transition is formulated by thermodynamics theory: the Gibbs free energy landscape can be effectively modulated under light, and the relative stability between different (meta‐)stable phases can be switched. This mechanism is applied to TMDs and reversible phase transitions between the topologically trivial 2H and nontrivial 1T′ phases are predicted, providing appropriate light frequency, polarization, and intensity are applied. The large energy barrier on the martensitic transformation path can be significantly reduced, yielding a small energy barrier phase transition with fast kinetics. Compared with other phase transition schemes, light illumination has great advantages, such as its non‐contact nature and easy tunability. The reversible topological phase transition can be applicable in high‐resolution fast data storage and in‐memory computing devices.

## Graphical abstract

Optomechanical theory is developed to predict how terahertz optics can drive ultrafast and reversible topological phase transitions in transition metal dichalcogenide monolayers, which is a non‐contacting approach that can work on free‐standing membranes. Density functional theory calculations are performed to evaluate the optical response functions based on photon–electron and photon–phonon coupling.

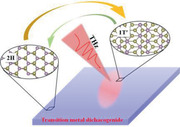

## 1 Introduction

The concept of topology in band structure has revolutionized condensed‐matter physics and materials science. Different phases of materials can be classified by their electronic band topological indices, which serve as indicators of various exotic properties.^[^ ^1^ ^]^ For example, the topologically protected surface/edge states of topological insulators (TIs) are promising platforms for fault‐tolerant quantum computing and high‐performance electronic or spintronic devices. Notably, some materials possess phases with contrasting topologies, and the topological phase transitions between these phases have evoked tremendous attention, owing to their scientific and technological importance.^[^ ^2^ ^]^

Until now, one of the most widely studied materials that could exhibit phases with contrasting topologies is the 2D group‐VI monolayer transition metal dichalcogenides (TMDs), in the chemical formula of MX_2_ (M = Mo and W, X = S, Se, and Te).^[^ ^3^ ^]^ Atomically, the M atom layer sits in the center, sandwiched by two X atom layers. Two (meta‐)stable phases, 2H and 1T′, could exist in TMDs. In the 2H phase, the three atom layers X‐M‐X show an A‐B‐A stacking pattern in a hexagonal lattice (space group of $P\bar{6}m2$, no. 187). If one X layer shifts about 1/3 lattice constant, the system would have an A‐B‐C stacking pattern, and is denoted as 1T phase. Such 1T phase is dynamically unstable and would spontaneously undergo a Peierls distortion, and finalizes to a distorted structure, 1T′ phase (space group of *P*2_1_/*m*, no. 11). Both 2H and 1T′ phases of monolayer group‐VI TMDs possess exotic properties. In the 2H phase, TMDs are semiconducting with large bandgaps (≈1 eV) and trivial electronic topology. Remarkably, the lack of inversion symmetry in the 2H phase induces an out‐of‐plane effective “magnetic” field, which lifts the spin degeneracy of both the valence and conduction bands. This leads to valley‐spin locking and a perfect valley contrasting circular dichroism.^[^ ^4^ ^]^ On the other hand, in the 1T′ phase, TMDs are theoretically predicted and experimentally demonstrated to be 2D *Z*_2_‐TIs,^[^ ^5^ ^]^ which exhibit topologically protected nontrivial edge states.

Both 2H and 1T′ TMDs are under intensive research. The transitions between these two phases are attracting particular attention. Technologically, the transition between 2H and 1T′ phases only requires a shuffle of one X layer. Thus, it is a crystalline‐to‐crystalline, diffusionless, and martensitic phase transition with much faster kinetics than diffusive phase changes (such as that in GeSbTe alloys^[^ ^6^ ^]^). Scientifically, such a phase transition could be an excellent platform to observe and investigate the process of trivial‐to‐nontrivial topological phase transitions. In addition, during the transition process, 1/6 of the M‐X chemical bonds need to break and reform (see below). Hence, the energy barrier is sufficiently high (over 1 eV per MX_2_),^[^ ^5a^ ^]^ and the two phases are fairly stable with long lifetime once a thorough phase transition occurs. Therefore, the monolayer TMDs and their phase transition could provide a good platform to observe and study quantum phase transition and martensitic phase transition in low‐dimensional materials, and may serve as potential data storage and in‐memory computing material.

However, the high energy barrier also makes it challenging to manipulate the different phases of monolayer TMDs. Energetically, 2H is usually more stable than 1T′ (except for monolayer WTe_2_). Previous theoretical works have predicted a few strategies to aid and facilitate phase transition from 2H to 1T′, including Li ion intercalation,^[^ ^7^ ^]^ mechanical tension,^[^ ^8^ ^]^ carrier injection,^[^ ^9^ ^]^ electron–hole pair excitation,^[^ ^10^ ^]^ and Ar‐plasma bombardment.^[^ ^11^ ^]^ Experimentally, visible laser irradiation on MoTe_2_ was proposed to achieve the 2H to 1T′ phase transition,^[^ ^12^ ^]^ but experiments thus far indicated that the new phase is a Te‐metalloid‐like phase, rather than 1Tʹ.^[^ ^13^ ^]^ Later, Wang et al. used ionic liquid gating to inject carriers into monolayer MoTe_2_ and observed a 2H to 1T′ transition.^[^ ^14^ ^]^ However, the phase transition easily reverses back once the gating is turned off. These facts also suggest that the phase transitions in monolayer MoTe_2_ is challenging. The metastable 1T′‐MoTe_2_ can be directly fabricated via chemical vapor deposition method^[^ ^15^ ^]^ by controlling Te vacancy concentration, but 1T′ to 2H phase transition could occur.^[^ ^16^ ^]^

**Figure 1:**
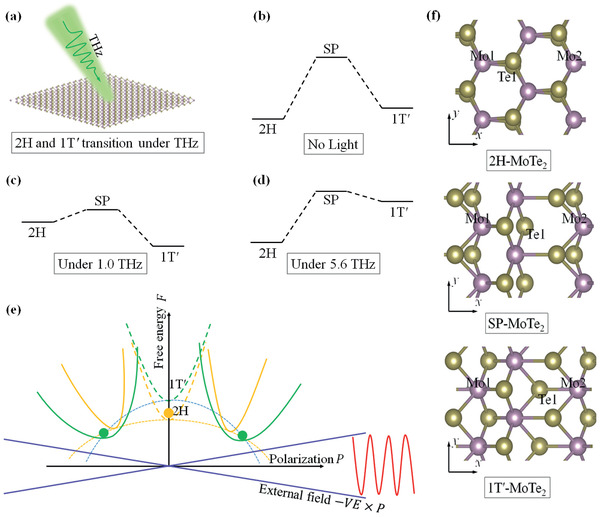
a) Schematic plot of THz irradiation on a monolayer 2H‐TMD, which could transform into 1T′‐TMD. b) Potential energy landscape of 2H, 1T′, and transition SP of TMD without THz irradiation. c) and d) are schematic energy profiles under different THz frequencies, which can well‐control the relative stability of the two phases. Note that the energy barrier could be significantly reduced (or even eliminated) if THz laser intensity, polarization, and frequency are appropriately selected. e) Gibbs free energy change under light illumination in the polarization (*P*) general coordinate system. Thick and long dashed orange and green curves represent intrinsic (no light irradiation) 2H and 1T′ structures, respectively. Under alternating THz illumination (red waves), electric field reduces Gibbs free energy (thin and short dashed curves), and the phase transition occurs (solid orange and green curves). f) Atomic geometries of 2H, SP, and 1T′ phases. Atoms in the 2H phase are slightly tilted for clarity reason. Note that even though the Mo1 and Mo2 atoms are periodic images, they possess different bonding nature with the lower Te layer (denoted as Te1). The armchair and zigzag directions are denoted as *x* and *y*, respectively.

Most of the above‐mentioned strategies require direct mechanical or electrochemical contacts between the sample and the tips or electrodes. Such contacts could introduce unwanted chemical impurities or additional interactions onto the monolayer TMDs; hence it may affect the sample quality and transition reversibility after many cycles.^[^ ^17^ ^]^ In the current work, we propose a new mechanism that is free of these problems. We theoretically predict that terahertz (THz) frequency laser can trigger ultrafast phase transitions between 2H and 1T′ (**Figure** **1**a). THz irradiation is a noncontact, noninvasive, athermic, and most‐often nondestructive technique,^[^ ^18^ ^]^ thus it could minimize lattice damages during operation.^[^ ^19^ ^]^ In addition, it can be deeper‐penetrating than visible lasers and the laser frequency, polarization, and intensity can be easily controlled, providing good flexibility. Here, we first theoretically demonstrate that under THz light irradiation, the potential energy landscape profile can be effectively altered, so that light irradiation would trigger phase transitions thermodynamically. To illustrate this theory, we apply first‐principles density functional theory (DFT, see Experimental Section) to evaluate the electron and phonon contributions to the optical susceptibility of monolayer TMDs in the THz range, and show that THz can effectively tune and flip relative stabilities between 2H and 1T′ phases (Figure 1b–d). In addition, the energy barrier on the phase transition path can be greatly reduced with well‐controlled THz frequency and intensity. In this case, ultrafast and reversible (2H↔1T′) topological phase transitions are expected. In this work, we mainly focus on monolayer MoTe_2_, because its energy difference between the 2H and 1T′ is the lowest among all monolayer TMDs. We also show that the THz driven phase transition is similarly applicable in other monolayer TMDs.

## 2 General Thermodynamic Theory

We will first illustrate our proposal with thermodynamic theory. The optical alternating electric field can be written as $\vec{E}\left(\omega ,t\right)=\vec{E}\;e^{i\omega t}$. In the low frequency range of interest (*ω* ≈THz), the wavelength of the light (a few hundred µm) is much larger than the dimension of a unit cell, therefore the optical electric field can be regarded as almost homogeneous (wavevector *k*≅0). For a 2D material or surface of a 3D material, when the light propagates along *z* (normal to the material surface), $\vec{E}$ is in the *xy*‐plane. Then, one could apply closed boundary condition^[^ ^20^ ^]^ and study the Gibbs free energy with the electric field $\vec{E}$ as external forcing

(1) $$G_{E}\;\left(A\right)=\min_{\delta \xi }\;\left\{F\left(\xi_{A}^{0}+\delta \xi \right)-\langle \vec{E}\cdot \vec{P}\left(\xi_{A}^{0}+\delta \xi \right)\rangle \right\}$$

Here *A* denotes the unperturbed structure with coordinates $\xi_{A}^{0}$, and it can be either 2H or 1T′ phases of TMD in our case. $\vec{P}\left(\xi \right)$ is the polarization of a formula unit. As the light is turned on, the system would interact with the light and $\vec{E}\cdot \vec{P}\left(\xi \right)$ is the work done. The new equilibrium coordinates should be $\xi_{A}^{0}+\delta \xi$, where the displacements *δξ* can be found by minimizing the Gibbs free energy. Note that *ξ* can be either ionic or electronic coordinates. Here we focus on ionic coordinates and the similar analysis applies to electronic coordinates as well. The polarization $\vec{P}\left(\xi \right)$ is related to *ξ* through the Born effective charges *Z* ^*^, and one has approximately $\vec{P}\;\left(\xi_{A}^{0}+\delta \xi \right)=\vec{P}\;\left(\xi_{A}^{0}\right)+Z^{*}\delta \xi$. Since for both phases that we are interested in (2H and 1T′), there is no permanent electric polarization $\vec{P}\left(\xi_{A}^{0}\right)$, thus $\vec{P}\;\left(\xi_{A}^{0}+\delta \xi \right)=Z^{*}\;\delta \xi$. So the work done $\vec{E}\cdot \vec{P}$(*ξ*) is linear in both $\vec{E}$ and *ξ*. On the other hand, the internal Helmholtz free energy *F* has leading positive‐quadratic dependence on *ξ*, which is roughly $F\;\left(\xi_{A}^{0}+\delta \xi \right)=\;F\left(\xi_{A}^{0}\right)+\frac{1}{2}\kappa {\left(\delta \xi \right)}^{2}$, where *κ* is the interatomic force constant. Such formalism also gives rise to the phonons. Then, the outcome of this standard linear‐quadratic minimization problem, to the leading order in *ξ* expansion, is

(2) $$\begin{array}{ccc} G_{E}\left(A\right) & = & \min_{\delta \xi }\left\{F\left(\xi_{A}^{0}+\delta \xi \right)-\vec{E}\cdot \vec{P}\left(\xi_{A}^{0}+\delta \xi \right)\right\}\;\;=\;F\left(\xi_{A}^{0}\right)\\ & & -\;\frac{1}{2}\langle \vec{E}\cdot \vec{P}\left(\xi_{A}^{0}+\delta \xi \right)\rangle \end{array}$$

which is illustrated in Figure 1e, the Gibbs free energy minimum position and value shifts linearly and quadratically with $\vec{E}$, respectively. Since the induced electric polarization is $\vec{P}=\varepsilon_{0}\;{\overset{↔}{\chi }}^{\prime }\cdot \vec{E}$, then its contribution to Gibbs free energy is $dG_{E}=-V_{f.u.}\varepsilon_{0}\vec{P}\cdot d\vec{E}=-V_{f.u.}\varepsilon_{0}\vec{E}\cdot {\overset{↔}{\chi }}^{\prime }\cdot d\vec{E}$. Integrating this over the electric field could give $\vec{E}\cdot \vec{P}\left(\xi_{A}^{0}+\delta \xi \right)=\frac{V_{f.u.}\varepsilon_{0}\vec{E}\cdot {\overset{↔}{\chi }}^{\prime }\left(\xi_{A}^{0},\;\omega \right)\cdot \vec{E}}{2}$, where $\varepsilon_{0}$ is the vacuum permittivity, *V*_f.u._ is the volume of the formula unit, and $\overset{↔}{\chi }\left(\xi_{A}^{0},\;\omega \right)$ is the susceptibility tensor of phase *A*. ${\overset{↔}{\chi }}^{\prime }\left(\xi_{A}^{0},\;\omega \right)$ is the real part of $\overset{↔}{\chi }\left(\xi_{A}^{0},\;\omega \right)$ (see Supporting Information for detailed discussions). The total Gibbs free energy is thus

(3) $$G_{E}\left(A,\omega \right)=F\left(\xi_{A}^{0}\right)-\frac{V_{f.u.}\varepsilon_{0}\vec{E}\cdot {\overset{↔}{\chi }}^{\prime }\left(\xi_{A}^{0},\;\omega \right)\cdot \vec{E}}{4}$$

Note that microscopically the electric field linearly interacts with the polarization, while the macroscopic Gibbs free energy depend on the $E^{2}$ (Equation (3)), which is schematically plotted in Figure 1e. Since neither 2H nor 1T′ phase contains an intrinsic in‐plane polarization, before light irradiation they both locate at $|\vec{P}|=\;0$. Here $\vec{P}$ serves as a generalized coordinate. When THz light is turned on, these two systems shift positions, depending on their optical susceptibility responses, and phase transition occurs when the 1T′ has lower Gibbs free energy than 2H. Note that if the system is in‐plane ferroelectric (with finite static polarization ${\vec{P}}_{s}$) and is under a slowly alternating electric field (very small *ω*), additional linear interaction term between ${\vec{P}}_{s}$ and $\vec{E}$ needs to be included.^21^ From thermodynamic laws, in equilibrium states the total Gibbs free energy $G_{E}\left(A,\omega \right)$ should be minimized with respect to different phases *A*. Without laser irradiation, only the intrinsic part $F_{0}\left(\xi_{A}^{0}\right)$ needs to be considered. For MoTe_2_, one has $F_{0}\left(\xi_{2H}^{0}\right)<F_{0}\left(\xi_{1T^{\prime }}^{0}\right)$, that is, 2H—MoTe_2_ is more stable than 1T′‐MoTe_2_. When an external laser is applied, the second term in (Eq. 3) comes into play. Through the dependence of ${\overset{↔}{\chi }}^{\prime }\left(\xi_{A}^{0},\omega \right)$ on $\xi_{A}^{0}$ (lowest order of $\frac{\partial {\vec{\chi }}^{\prime }\left(\xi ,\omega \right)}{\partial \xi }$), $G_{E}\left(A,\omega \right)$ could have different shape from $F_{0}\left(\xi_{A}^{0}\right)$, and the relative stability between different phases can be switched. Note that $\frac{\partial {\overset{↔}{\chi }}^{\prime }\left(\xi ,\omega \right)}{\partial \xi }$ corresponds to the Raman scattering process, during which photons from the laser field excites coherent phonons in the material system, and the material is thus driven out of equilibrium. As a result, with selected laser polarization, frequency, and intensity, an inverted order $G_{E}\left(1T^{\prime },\;\omega \right)<G_{E}\left(2H,\;\omega \right)$ could be achieved. This indicates a topological phase transition under THz irradiation.

At the THz regime, the optical susceptibility tensor contains contributions from both electron and ion (phonon) subsystems, $\overset{↔}{\chi }\;\left(\omega \right)={\overset{↔}{\chi }}^{\mathrm{el}}\left(\omega \right)+{\overset{↔}{\chi }}^{\mathrm{ph}}\left(\omega \right)$, and the ${\overset{↔}{\chi }}^{\mathrm{el}}\left(\omega \right)$ and ${\overset{↔}{\chi }}^{\mathrm{ph}}\left(\omega \right)\;$can be evaluated separately.

## 3 Results and Discussion

### 3.1 DFT Simulation Results on MoTe_2_

The transition saddle point (SP) structure of MoTe_2_ is obtained through cell‐variable nudged‐elastic band calculation. Geometrically, it almost remains the rectangular shape unit cell, and keeps the *M_y_* mirror symmetry as in both 2H and 1T′ phases. Compared with the 2H phase, which is the starting point of the phase transition, the transition SP lattice is elongated in the *x*‐direction by 4% and shrinks in the *y*‐direction by 5%, toward the 1T′ phase. As for the internal chemical bonding structure, we denote the relevant atoms as Mo1, Mo2, and Te1, as indicated in Figure 1f. Here the Te1 refers to the Te atom on the lower Te layer, and the Mo1 and Mo2 are periodic images of a single Mo atom in the simulation supercell (containing 2 Mo and 4 Te atoms). One could clearly see that from the 2H phase to the transition SP, the main atomic displacement is the movement of Te1 along the +*x* direction, and the chemical bond Mo1‐Te1 breaks. Hence, the total energy of the system increases as the system approaches the SP. The Mo1 (Mo2) correspondingly moves to +*x* (−*x*), but the displacements are much smaller. After passing the transition state, the Te1 forms chemical bond with Mo2 and the system becomes 1T′ phase. This reduces the total energy. Therefore, during the whole process, 1/6 chemical bonds (one Mo‐Te bond among all six bonds) in the system break and reform, which makes the energy barrier high enough to protect the 2H and 1T′ phases under ambient environment.

**Figure 2:**
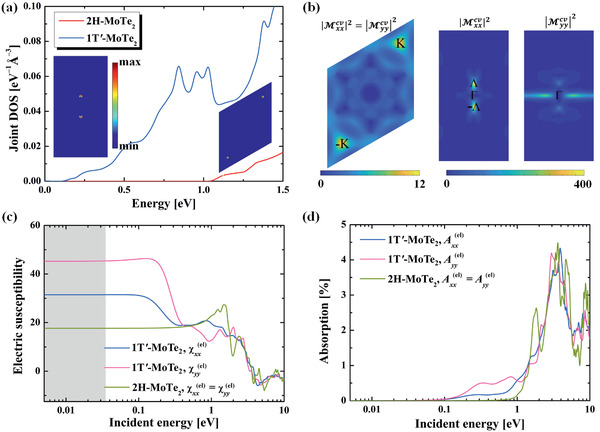
a) jDOS of monolayer 2H and 1T′ MoTe2 as a function of incident photon energy ℏ*ω*. Insets show the ***k***‐resolved jDOS of 2H‐MoTe_2_ at ℏ*ω* = 1.2 eV and of 1T′‐MoTe_2_ at ℏ*ω* = 0.2 eV. b) ***k***‐resolved transition dipole, in Å^2^. c) The real part of electron contribution to the electric susceptibility and d) absorbance of 2H and 1T′ MoTe_2_ under different LPL illumination. The gray shaded area in c) is the phonon frequency regime (0−8 THz).

Now we are ready to apply the above theory to monolayer MoTe_2_ under linearly polarized terahertz laser (LPTL). According to Equation (3), we calculate the susceptibility $\overset{↔}{\chi }\left(\omega \right)$ at THz frequencies, which is contributed by both electron and ion subsystems. First, we explore the electron subsystem. The calculated electron band dispersions are shown in Supporting Information. One knows that the photon–electron interaction is mainly determined by two factors, namely, the joint density of states (jDOS) and the transition dipole matrix. The jDOS reads

(4) $$j\left(\hbar \omega \right)=\frac{1}{{\left(2\pi \right)}^{3}}\;\int_{\mathrm{BZ}}\sum_{c,v}\delta \left(\varepsilon_{ck}-\varepsilon_{vk}-\hbar \omega \right)dk$$

where the $\varepsilon_{nk}$ represents the energy of band‐*n* at momentum ***k***. *c* and *v* denote conduction and valence bands, respectively. The integral is taken in the first Brillouin zone (BZ). The jDOS of 2H and 1T′‐MoTe_2_ are plotted in **Figure** **2**a. One clearly sees that the 2H—MoTe_2_ possesses a much smaller jDOS than that of 1T′‐MoTe_2_ for ℏ*ω* < 1.5 eV, suggesting weaker optical response. We also plot the ***k***‐resolved jDOS (the integrand in Equation (4)) for ℏ*ω* slightly above the direct bandgap of the two phases [at ℏ*ω* = 1.2 eV (0.2 eV) for 2H (1T′)‐MoTe_2_]. It can be observed that states around ± K (± Λ) points in the BZ dominates the jDOS of 2H (1T′)‐MoTe_2_. Here ±Λ points are locations of the Dirac points of 1T′‐MoTe_2_ before spin‐orbit coupling is included.

We then examine the transition dipole matrix between the valance and conduction bands, $|M_{\mathrm{ii}}^{\mathrm{cv}}\left(k\right){|}^{2}=|\langle u_{ck}|\nabla_{k_{i}}\left|u_{vk}\right\rangle {|}^{2}$, where |*u_n_*_***k***_〉 is the periodic part of Bloch wavefunction. For 2H–MoTe_2_ (left panel of Figure 2b), the transition dipole has a maximum value of 12 Å^2^ around ±K and an average value of 2.4 Å^2^ over the first BZ. On the contrary, for 1T′‐MoTe_2_, the transition dipoles are significantly larger (middle and right panels of Figure 2b). One can see $|M_{\mathrm{xx}}^{\mathrm{cv}}\left(k\right){|}^{2}$ is mainly contributed from the states around ±Λ, with a maximum value of 386 Å^2^. $|M_{\mathrm{yy}}^{\mathrm{cv}}\left(k\right){|}^{2}$ have major contributions from both ±Λ and the −X ↔ +X path, with a maximum value of 260 Å^2^. Here *x* and *y* directions are perpendicular to and parallel with the dimerized Mo chain in the 1T′ structure, respectively. The average value of $|M_{\mathrm{xx}}^{\mathrm{cv}}\left(k\right){|}^{2}$ and $|M_{\mathrm{yy}}^{\mathrm{cv}}\left(k\right){|}^{2}$ of 1T′‐MoTe_2_ in the first BZ are 5.4 and 9.1 Å^2^, respectively. These suggest that the optical responses of 1T′‐MoTe_2_ should be stronger than that of 2H‐MoTe_2_, consistent with previous studies that TIs have enhanced optical responses than normal insulators, due to band mixing between the chalcogen p‐band and transition‐metal d‐band.^[^ ^22^ ^]^ In addition, 1T′‐MoTe_2_ should have stronger optical response to the *y*‐polarized LPTL than to the *x*‐polarized LPTL.

The arguments above are verified by direct calculations of the susceptibility of 2H and 1T′‐MoTe_2_. According to random phase approximation, the electron contributed susceptibility can be expressed as^[^ ^23^ ^]^

(5) $$\chi_{\mathrm{ii}}^{\mathrm{el}}\;\left(\omega \right)=\;-\frac{e^{2}}{\varepsilon_{0}}\int_{\mathrm{BZ}}\frac{d^{3}k}{{\left(2\pi \right)}^{3}}\sum_{n,m}\frac{\left(f_{nk}-f_{mk}\right){\left|M_{\mathrm{ii}}^{\mathrm{nm}}\left(k\right)\right|}^{2}}{\varepsilon_{nk}-\varepsilon_{mk}-\hbar \omega -i\eta }$$

where $f_{nk}={\left[1+\exp\frac{\left(\varepsilon_{nk}-E_{F}\right)}{k_{B}T}\right]}^{-1}\;$is the Fermi–Dirac electron occupancy of band‐*n* at ***k***, with *E*_F_ as the Fermi level. In our simulation, we take *T* = 300 K and the phenomenological damping parameter *η* = 0.025 eV. The calculated $\chi_{ii}^{\mathrm{el}}\left(\omega \right)$ is a complex function, $\chi_{ii}^{\mathrm{el}}\;\left(\omega \right)=\chi_{ii}^{{}^{\prime },\mathrm{el}}\;\left(\omega \right)+i\chi_{ii}^{{}^{\prime \prime },\mathrm{el}}\left(\omega \right)$. For a 2D material computed with a 3D periodic supercell, the artificial contribution from vacuum space along the *z*‐direction needs to be eliminated. In practice, the integration over the BZ is carried out by a ***k*** ‐mesh sampling, $\int_{\mathrm{BZ}}\frac{d^{3}k}{{\left(2\pi \right)}^{3}}=\frac{1}{V}\;\sum_{k}w_{k}=\frac{1}{\mathrm{Sh}}\;\sum_{k}w_{k}$, where *S* is in‐plane (*x*‐*y* plane) area, *h* is the thickness of the 3D simulation supercell, and *w_**k**_* is the weight of each ***k*** point. For the in‐plane components of the susceptibility tensor, according to a parallel capacitor model^[^ ^24^ ^]^ one could use a scaling relationship $\chi_{ii}^{{}^{\prime },\mathrm{sc}}\left(\omega \right)h\;=\chi_{ii}^{{}^{\prime },2D}\;\left(\omega \right)d$ to eliminate the influence of the artificial thickness *h*. Here $\chi_{ii}^{{}^{\prime },\mathrm{sc}}\left(\omega \right)$ and $\chi_{ii}^{{}^{\prime },2D}\left(\omega \right)$ are supercell calculated values and the rescaled values of the real part of the susceptibility tensor, respectively. *d* is an effective thickness of the 2D material, which is taken to be the distance between the centers of two layers when the same 2D material is van der Waals stacked in its bulk phase. Note that this *d* is a phenomenological quantity (similar as gauge choice in field theory). Fortunately, when we apply Equation (3) to estimate the LPTL‐induced Gibbs free energy, the susceptibility is multiplied with the total volume *V*_f.u._. Hence, the final results are independent of both *h* and *d*. As for the calculated imaginary part $\chi_{ii}^{{}^{\prime \prime }}\left(\omega \right)$, it reflects the optical absorbance $A_{ii}\;\left(\omega \right)=\;1-\exp\left[-\frac{\omega }{c}\chi_{ii}^{{}^{\prime \prime },\mathrm{sc}}\left(\omega \right)h\right]$, where *c* is speed of light in vacuum.

We plot the rescaled (with *d* = 7.7 Å) real part of electron contributed susceptibility and absorbance in Figure 2c,d. One observes that the absorbance of 2H‐MoTe_2_ starts at ≈1 eV, owing to its large direct bandgap at ±K points. Whereas in 1T′‐MoTe_2_, the absorbance occurs at ≈0.1 eV due to its smaller direct bandgap. The absorbance for the *y*‐LPTL is larger than that for the *x*‐LPTL, consistent with previous analysis based on the transition dipole matrix. According to the Kramers–Kronig relation, if the absorbance occurs at lower energy with larger value, the real part susceptibility would be higher at low frequency. This agrees with our numerical results. One sees that at low‐frequency regime (e.g., THz, gray shaded areas in Figure 2c), $\chi_{xx}^{{}^{\prime },\mathrm{el}}\;\left(2H,\omega \right)=\chi_{yy}^{{}^{\prime },\mathrm{el}}\;\left(2H,\;\omega \right)=\;17.7$, while $\chi_{xx}^{{}^{\prime },\mathrm{el}}\;\left(1T^{\prime },\;\omega \right)=\;31.5$ and $\chi_{yy}^{{}^{\prime },\mathrm{el}}\;\left(1T^{\prime },\;\omega \right)=\;45.2$. Since no photon absorption (electron–hole pair generation) occurs in the THz regime, the real parts of susceptibilities keep almost constant.

Next, we analyze the phonon contribution to the susceptibility. According to lattice dynamics theory and group theory analysis, only IR‐active vibration modes can directly interact with LPTL and contribute to the first‐order susceptibility. Also, since the wavevector of photons are much smaller than the dimensions of the BZ, only phonons near Γ‐point need to be considered. We calculate the phonon dispersion (see Supporting Information), and label the optical phonon modes according to the irreducible representations of the corresponding crystalline symmetry group. For the 2H phase, one has $\Gamma_{2H}=E^{\prime }\;⊕A_{1}^{\prime }⊕E^{\prime \prime }⊕A_{2}^{{}^{\prime \prime }}$ and only the two dimensional *E*′ mode is IR‐active. Since the 2H phase is inversion asymmetric, this *E*′ mode would split into LO and TO modes, where the LO mode contributed to optical responses. For the 1T′ phase, one has Γ_1T′_ = 6*A_g_*⊕3*B_g_*⊕2*A_u_*⊕4*B_u_* and the one dimensional *B_u_* and *A_u_* modes are IR‐active under the *x*‐ and *y*‐LPTL, respectively.

**Figure 3:**
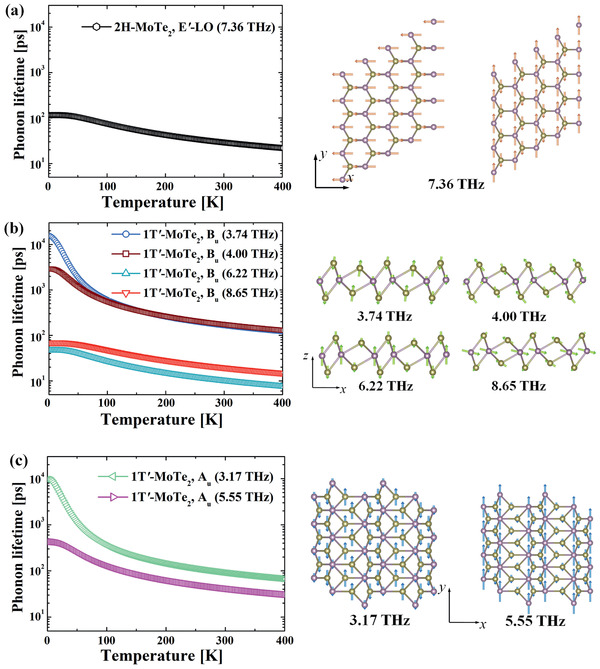
Near Γ‐point IR‐active phonon mode lifetime variation as a function of temperature of a) 2H‐MoTe_2_, b) *x*‐polarized in 1T′‐MoTe_2_, and c) *y*‐polarized in 1T′‐MoTe_2_. The vibrational modes are plotted in the right panel. Note that the two modes in (a) are near Γ‐point modes along *x* and *y* directions (*k_x_*→0 and *k_y_*→0).

The phonon contributed susceptibility can be calculated via^[^ ^25^ ^]^

(6) $$\chi_{ij}^{\mathrm{ph}}\;\left(\omega \right)=\frac{1}{V}\sum_{m}\frac{S_{m,ij}}{\omega_{m}^{2}-{\left(\omega +i\tau_{m}^{-1}\right)}^{2}}$$

where $S_{m,ij}=\left[\sum_{\mu ,k}Z_{\mu ,ik}^{*}u_{m}\left(\mu k\right)\right]\;\left[\sum_{\mu^{\prime },k^{\prime }}Z_{\mu^{\prime },jk^{\prime }}^{*}u_{m}\left(\mu^{\prime }k^{\prime }\right)\right]$ is the oscillator strength of the *m*‐th phonon mode (*i*,*j* = *x*,*y*). Here $Z_{\mu ,ik}^{*}$ is the Born effective charge on ion‐*μ*, and *u_m_*(*μk*) is mass‐normalized displacement of the *m*‐th mode on the *μ*‐th ion in the *k*‐th direction. *ω_m_* and *τ_m_* are the frequency and lifetime of the near Γ‐point phonons. The phonon lifetime comes into play because it influences the photon absorption linewidth and strength by IR‐active phonons. When the frequency of the photon is close to that of a phonon, the photon can be absorbed by the phonon and then later dissipated into heat. According to the uncertainty principle, the absorption peak frequency and linewidth are *ω_m_* and 1/*τ_m_*, respectively. In other words, the photon absorption mainly occurs in the frequency regime between *ω_m_*−1/*τ_m_* and *ω_m_*+1/*τ_m_*. Out of this window, photons are unlikely to be directly absorbed by the system (*χ*′′^,ph^ ≅0). In this case, the photons can be treated as a pure alternating electric field with small dissipation. The phonon lifetime is dominated by three‐phonon scattering (see Supporting Information), which originates in the third‐order anharmonicity of the atomic potential. In **Figure** **3** we plot our calculated lifetime (*τ_m_*) of IR‐active phonon modes. As temperature rises, the three‐phonon scattering accelerates because more phonons are excited, thus phonon lifetime reduces. In detail, the lifetime of *E*′‐LO mode in 2H—MoTe_2_ is on the order of 10^1^ ps at room temperature, suggesting a linewidth of the order of 0.1 meV. For 1T′‐MoTe_2,_ the low frequency *B_u_* and *A_u_* modes (below 5 THz) has a much longer lifetime (thus a smaller linewidth).

Besides lifetime, another important factor that influences the phonon contributed susceptibility is the pattern of the ion displacements of vibrational modes. For example, a phonon can contribute to $\chi_{xx}^{\mathrm{ph}}\left(\omega \right)$ only when it is IR‐active, and it vibrates strongly along the *x*‐direction. The vibrational displacements of IR‐active modes are also plotted in Figure 3b. One sees that the *E*′‐LO of 2H phase has two 90°‐rotation related degenerate modes that could couple to *x*‐ and *y*‐LPTL. Three *B_u_* modes (at 3.74, 4.00, and 6.22 THz) of the 1T′ phase have small *x*‐direction but large *z*‐direction vibration components, so they do not contribute much to $\chi_{xx}^{\mathrm{ph}}\left(\omega \right)$. Only the *B_u_* mode at 8.65 THz vibrates strongly along *x*. The *A_u_* mode of the 1T′ phase at 3.17 THz vibrates along both *y* and *z*, while the mode at 5.55 THz is mainly along *y*.

**Figure 4:**
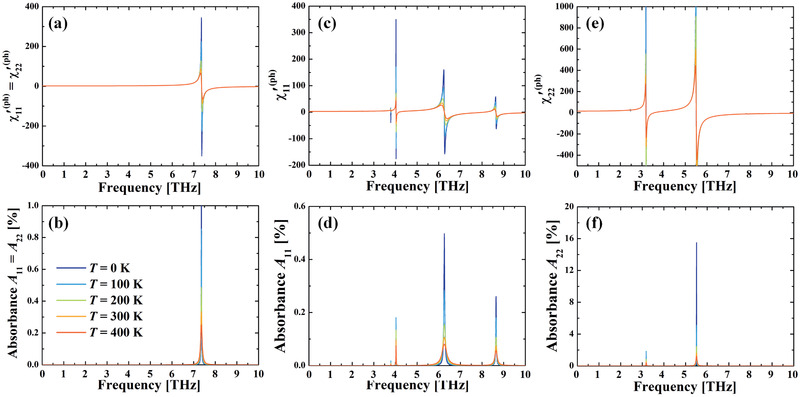
IR phonon modes contributed frequency‐dependent electric susceptibility function of a,b) 2H—MoTe_2_, c,d) 1T′‐MoTe_2_ under *x*‐LPTL, and e,f) 1T′‐MoTe_2_ under *y*‐LPTL. Here (a), (c), and (e) are real part of susceptibility, and (b), (d), and (f) are absorbance. Modes lifetime under different temperatures are plotted.

We can calculate the phonon contributed susceptibility. Note that the phonon lifetime is temperature dependent. Hence, we plot the results under several selected temperatures, in **Figure** **4**. It is clearly seen that the imaginary parts are composed of Lorentzian‐shape peaks sitting on the frequencies of IR‐active phonon modes. As temperature increases, the absorption peaks get shorter and wider because the phonon lifetime reduces (linewidth increases). For the real part of susceptibility, such temperature variation only affects their values near the resonant regime (|*ω* − *ω_m_*| ≲ 1/*τ_m_*). Out of this regime (|*ω* − *ω_m_*| ≳ 1/*τ_m_*), the real part susceptibility response remains nearly unchanged as temperature varies. At low frequency (lower than first absorbance peak), the calculated susceptibilities are $\chi_{xx}^{{}^{\prime },\mathrm{ph}}\;\left(2H,\omega \right)=\chi_{yy}^{{}^{\prime },\mathrm{ph}}\;\left(2H,\omega \right)=\;1.8$ and $\chi_{xx}^{{}^{\prime },\mathrm{ph}}\;\left(1T^{\prime },\;\omega \right)=\;2.9$ and $\chi_{yy}^{{}^{\prime },\mathrm{ph}}\;\left(1T^{\prime },\;\omega \right)=\;15.4$. According to the Kramers–Kronig relation, near the absorption peak, the real parts of the susceptibility is characterized by a sharp jump between positive and negative values, which are clearly seen in Figure 4. When the phonon linewidth is small (on the order of 0.01 THz), the absolute value of *χ* ^′,ph^ can be as large as a few hundred around the absorption peak. This offers us opportunities to manipulate the potential energy landscape effectively when the THz frequency is carefully selected, according to Equation (3).

**Figure 5:**
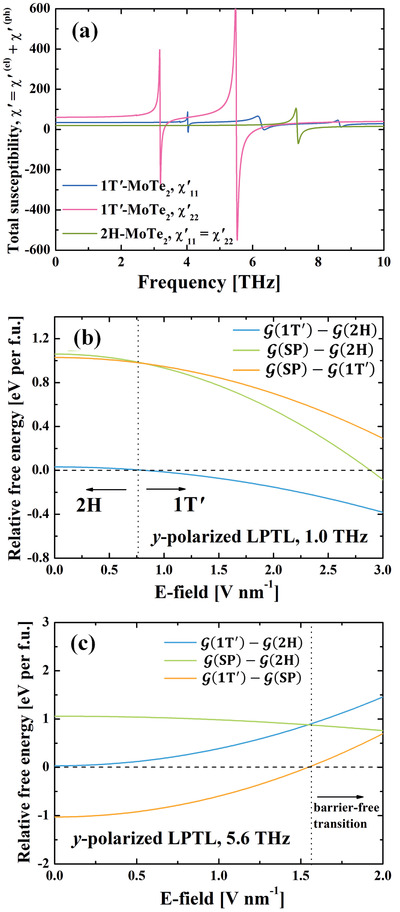
a) Total (electron and phonon) real part of the electric susceptibility of monolayer MoTe_2_. Room temperature phonon lifetime is taken here. Relative Gibbs free energy between different states under a *y*‐LPTL with frequency b) 1.0 THz and c) 5.6 THz.

Now we add the electron and phonon contributed susceptibilities together (**Figure** **5**a). One can see that for *ω* < 3 THz, the maximum values of $\chi_{yy}^{\prime }\left(1T^{\prime },\;\omega \right)$ and $\chi_{xx}^{\prime }\left(1T^{\prime },\;\omega \right)$ are 60.9 and 34.5, respectively. While for 2H‐MoTe_2_, one has $\chi_{xx}^{\prime }\;\left(2H,\;\omega \right)=\chi_{yy}^{\prime }\;\left(2H,\omega \right)=\;19.5$. If one uses a *y*‐LPTL of *ω* = 1 THz, the susceptibility difference between the 1T′‐MoTe_2_ and 2H—MoTe_2_ is then 41.4. Hence, from Equation (3), such a laser tends to push the system toward 1T′ phase and stabilize it. When the laser intensity in free space *I* reaches 8.6 × 10^10^ W⋅cm^−2^ (*E* = 0.80 V⋅nm^−1^, from $I=\frac{1}{2}\varepsilon_{0}cE^{2}$), the 1T′‐MoTe_2_ would have lower Gibbs free energy than that of 2H‐MoTe_2_, as shown in Figure 5b. This would lead to a thermodynamic phase transition from 2H to 1T′. If we move closer to the frequencies of IR‐active phonon modes of 1T′‐MoTe_2_, the susceptibility difference between 2H and 1T′ phases would dramatically increase. For example, at *ω* = 5.45 THz, $\chi_{yy}^{\prime }\left(2H,\;\omega \right)$ and $\chi_{yy}^{\prime }\left(1T^{\prime },\;\omega \right)\;$are 21.5 and 519.0, respectively. Hence a *ω* = 5.45 THz *y*‐LPTL with its laser intensity as small as 7.6 × 10^9^ W⋅cm^−2^ (*E* = 0.24 V⋅nm^−1^) is sufficient to make the 1T′ phase more stable than 2H. Our result agrees well with the recent experimental observations^[^ ^26^ ^]^ that a 0.3−1.5 THz (centered at 0.5 THz) irradiation could drive a 2H→1T′ phase transition in monolayer MoTe_2_. The free space field amplitude used in the experiment is 0.03 V⋅nm^−1^, which is further field enhanced by 20−50 times in most regions; hence the field amplitude irradiated onto the samples is around 0.6−1.5 V⋅nm^−1^, consistent with our estimates.

The above prediction suggests that *y*‐LPTL irradiation triggers ion movement along *x*, which is the 2H→1T′ structural change path, which is counterintuitive. In order to confirm and visualize this effect in real space, we perform an ab initio molecular dynamics simulation by applying an alternating electric field along *y*. The simulation results clearly indicate an ion equilibrium position movement along *x*. Around the new equilibrium state, the ions vibrate with *ω_m_* = 7.1 THz (see Supporting Information for details). These are consistent with macroscopic thermodynamic theory prediction.

### 3.2 Thermal Effect under THz

We estimate the temperature rise of the system under THz irradiation. When the laser frequency is chosen out of direct photon absorbance regime (*χ*′′≅0), the energy transfer from photon absorption can be omitted. We only consider optical alternating electric field effect here. First, before phase transition, the system oscillates according to the driven oscillator equation

(7) $$\frac{d^{2}x_{\mu }}{dt^{2}}+2\zeta \omega_{m}\frac{dx_{\mu }}{dt}+\omega_{m}^{2}\;x_{\mu }=\frac{1}{m_{\mu }}\;E_{0}^{y}Z_{\mu ,xy}^{*}\cos\omega t$$

where *x_µ_*, *m_µ_*, and $Z_{\mu ,xy}^{*}$ are average values of displacement, mass, and Born effective charge of ion‐*μ*. *ω_m_* = 7.1 THz and *ζ* are the phonon frequency and its effective damping, and *E*_0_ = 1 V⋅nm^−1^ and *ω* = 1 THz are the THz light magnitude and frequency, respectively. The displacement amplitude is then

(8) $$x_{\mu }^{\max}=\frac{Z_{\mu ,xy}^{*}E_{0}^{y}}{m_{\mu }\omega \sqrt{{\left(2\omega_{m}\zeta \right)}^{2}+{\left(\frac{\omega_{m}^{2}-\omega^{2}}{\omega }\right)}^{2}}}$$

The amplitudes of Mo and Te are estimated to be 0.0085 and 0.0031 Å, respectively, well consistent with ab initio molecular dynamics simulation results (Supporting Information). We use the heat capacity of MoTe_2_ (18.4 cal⋅K^−1^⋅mol^−1^)^[^ ^27^ ^]^ and estimate the temperature rise when these driven oscillating motions fully convert into heat, $c_{p}T\;=\frac{1}{2}\;\sum_{\mu }m_{\mu }\omega_{m}^{2}{\left(x_{\mu }^{\max}\right)}^{2}$, which gives 6 K.

In addition, the work done under THz irradiation during phase transition could also dissipate into heat, which is $W=\frac{1}{4}V_{f.u.}\varepsilon_{0}\left(\chi_{1T^{\prime }}-\chi_{2H}\right)E_{0}^{2}$. The temperature rise from this source is similarly estimated to be 8 K. Therefore, during THz driven phase transition of MoTe_2_, the temperature rise effect is small enough and can be dissipated out quickly. The waste heat problem in conventional phase change materials can be eliminated here.

### 3.3 Reduced Energy Barrier Phase Transition

In order to trigger fast phase transitions, one has to evaluate the kinetics along the transition path. We use the SP structure and its optical responses (see Supporting Information) to estimate the energy barrier change under LPTL. Without laser irradiation, the energy barrier from 2H to 1T′ is found to be 1.06 eV per f.u. (corresponding to 160 µJ⋅cm^−2^). We find that in the THz range, the electron contributed susceptibility of the SP is $\chi_{xx}^{{}^{\prime },\mathrm{el}}\;\left(\mathrm{SP},\;\omega \right)=\;129.0$ and $\chi_{yy}^{{}^{\prime },\mathrm{el}}\;\left(\mathrm{SP},\;\omega \right)=\;122.0$. The phonon contribution to the susceptibility is also evaluated. At 1 THz, the $\chi_{xx}^{{}^{\prime },\mathrm{ph}}=\;13.0$ and $\chi_{xx}^{{}^{\prime },\mathrm{ph}}=\;14.2$. The total susceptibility of the transition SP structure is plotted in Supporting Information. Hence, even though a *y*‐LPTL with *ω* = 1 THz and *E* = 0.80 V⋅nm^−1^ is large enough to induce a phase transition from 2H to 1T′ thermodynamically, according to Equation (3), the energy barrier is still 0.97 eV per f.u. (corresponding to 145.5 µJ⋅cm^−2^). One can increase its intensity to *I* = 1.1 × 10^12^ W⋅cm^−2^ (*E* = 2.84 V⋅nm^−1^), and the energy difference between the SP and the 2H phase may be wiped out. However, thorough evaluation the free energy landscape under THz irradiation is a high dimensional problem. Here, this Gibbs free energy comparison is a simple estimate. Nevertheless, we expect that under this THz intensity, the energy barrier can be significantly reduced. According to the Arrhenius law, phase transition kinetics exponentially increases as the energy barrier reduces, hence it corresponds to a fast transition under such intense light irradiation. Considering the large contrasting optical susceptibilities between these phases, it is possible to erase the energy barrier, once the optical frequency and intensity are carefully selected, but an exact prediction is very challenging. If the energy barrier is completely erased, the topological phase transition could happen on the timescale of a few picoseconds.^[^ ^28^ ^]^ Even in this situation, the system needs additional time to dissipate its kinetic energy and fully relax to the new equilibrium state (1T′), which could occur on the order of tens of picoseconds.^[^ ^29^ ^]^ Note that this is a rough estimate, and the dissipation time also depends on the boundary condition of the material. In addition, one has to note that our model is based on a unit cell calculation, which corresponds to a coherent phase transition in the whole sample. In reality, the material may contain different domains, and the domain boundary energy (on the order of a few tens of meV⋅Å^−1^)^[^ ^8^ ^]^ could change the free energy profile. Then the nucleation barrier associated with the domain boundary needs to be overcome during phase transition. In order to further explore the phase transition kinetics and its relationship with light frequency and strength, more experiments with stringent condition need to be conducted. In the recent experiment,^[^ ^26^ ^]^ the field acting onto the sample is around 0.6–1.5 V⋅nm^−1^. According to our model, this can still have an energy barrier, hence, a transition timescale on the order of nanosecond, as observed in the experiment, is quite reasonable.

Even though the 2H phase is energetically more stable without any external stimulus, once a large area of 1T′ phase is fabricated, it is difficult to overcome the high energy barrier and transit back to 2H. This non‐volatile phase transition guarantees data safety for long‐term storage. As for data writing, one needs another stimulus to boost a 1T′→2H phase transition. This can be done by selecting the frequency regime where *χ*′(2H, *ω*) is much larger than *χ*′(1T′, *ω*). From Figure 5a, one sees that a *y*‐LPTL with *ω* = 5.63 THz can be used. This is slightly above the IR‐active mode of 1T′‐MoTe_2_ at *ω* = 5.55 THz. At this frequency, $\chi_{yy}^{\prime }\left(2H,\;\omega \right)$, $\chi_{yy}^{\prime }\left(\mathrm{SP},\;\omega \right)$, and $\chi_{yy}^{\prime }\left(1T^{\prime },\;\omega \right)$ are 21.5, 89.9, and −305.1, respectively. Hence, as the *y*‐LPTL intensity increases, the 2H and 1T′ energy difference is also enhanced (Figure 5c). Particularly, a LPTL with *I* = 3.0 × 10^11^ W⋅cm^−2^ (*E* = 1.53 V⋅nm^−1^) is sufficient to greatly reduce the transition energy barrier. This completes the ultrafast topological phase transition cycle (2H↔1T′) in monolayer MoTe_2_. The optical susceptibility and Gibbs free energy for typical frequencies and electric field strengths are listed in **Table** **1**.

**Table 1:** Lattice constant, relative energy, optical susceptibility, and the relative Gibbs free energy under *y*‐polarized LPTL for the 2H, transition SP, and 1T′ phases of monolayer MoTe_2_

Monolayer MoTe_2_	2H	SP	1T′
Lattice constant (unit cell) [Å]	*a* = 3.49	*a* = 6.30	*a* = 6.31
	*b* = 3.49	*b* = 3.29	*b* = 3.37
Relative energy [eV per f.u.]	0.00	1.06	0.03
Total susceptibility (1 THz)	19.5	136	60.9
Relative free energy [eV per f.u.] (1 THz, *E* = 0.80 V⋅nm^−1^)	0.00	0.97	0.00
Total susceptibility (5.45 THz)	21.5	68.9	519
Relative free energy [eV per f.u.] (5.45 THz, *E* = 0.24 V⋅nm^−1^)	0.00	1.05	0.00
Total susceptibility (5.63 THz)	21.5	89.9	−305
Relative free energy [eV per f.u.] (5.63 THz, *E* = 1.53 V⋅nm^−1^)	−0.89	0.00	0.00

2D materials with atomic‐scale thickness in the out‐of‐plane direction may reduce the data unit volume (*L* × *L* × *d* in the real space), compared with conventional 3D materials (*L* × *L* × *L*). Here *L* is characteristic lateral size (usually a few tens to a few hundred nanometers) and *d* is effective thickness (sub‐nanometer). In addition, the topological metallic edge state in 1T′ TMD monolayers is a few nanometers,^[^ ^5a^ ^]^ which allows minimum horizontal feature size to be a few tens nanometers. In this way, the single data storage unit of 2D monolayer MoTe_2_ may occupy smaller space than 3D bulk materials, and the information storage density (over the real space) can be high. Since the 2H‐MoTe_2_ is a semiconductor with sizable bandgap, while the 1T′‐MoTe_2_ has a small topological bandgap, the electric resistance and optical reflection could be highly distinct in the two phases.^[^ ^30^ ^]^ Thus, one may use electrical or optical approach to distinguish the 2H and 1T′ phases. If the sample quality is good and the phase transition occurs completely, the data reading resolution should be high enough for practical applications. We thus propose that the monolayer MoTe_2_ can be used as potential memory devices with large information storage density.

### 3.4 Other Monolayer TMD Systems

**Figure 6:**
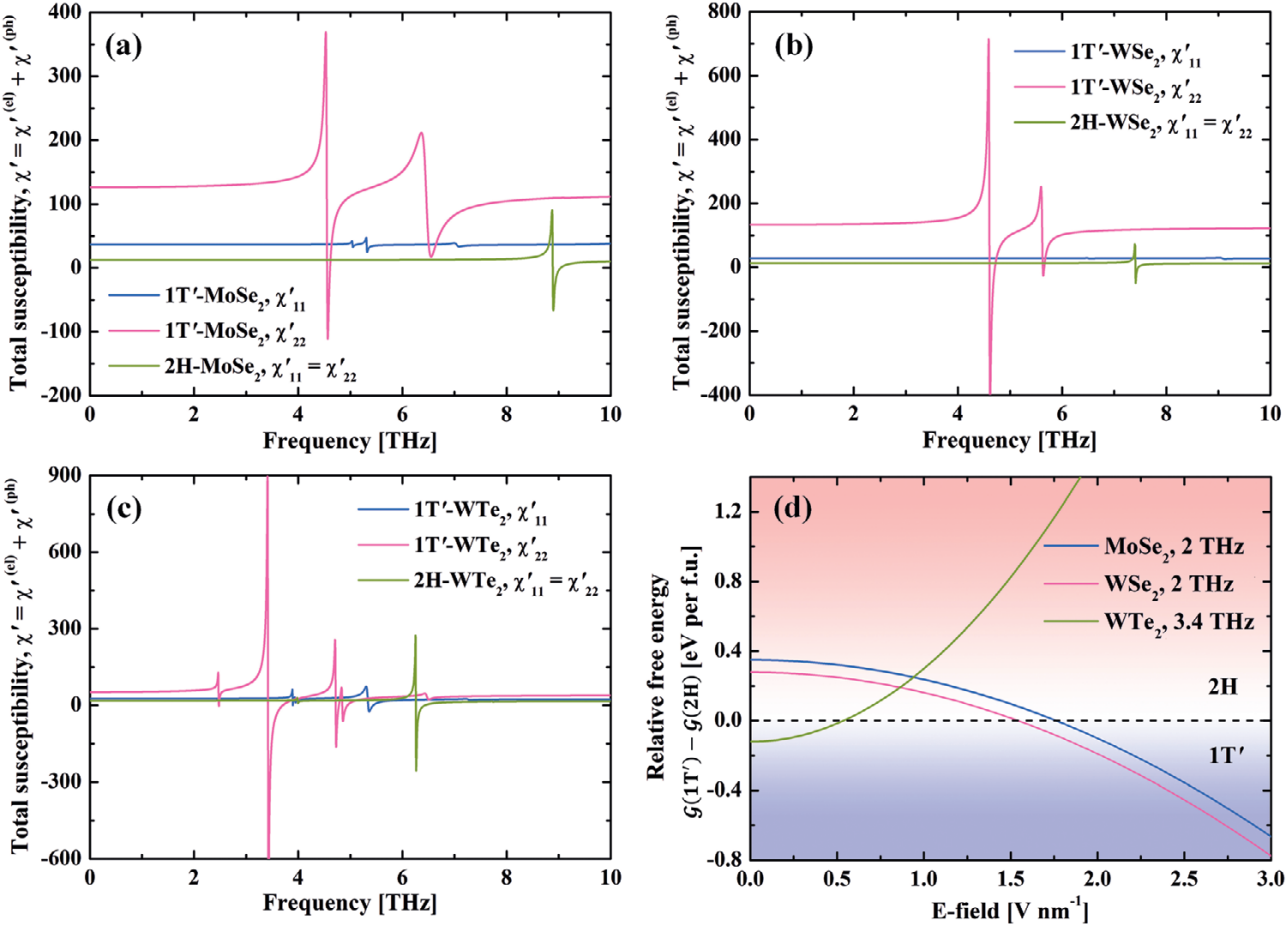
Total (electron and phonon) real part of susceptibility of a) MoSe_2_, b) WSe_2_, and c) WTe_2_ under LPTL. Phonon lifetime at room temperature is taken. d) Gibbs free energy difference between the 2H and 1T′ phases under (*y*‐polarized) THz irradiation. The positive and negative energy values represent that the 2H and 1T′ is more stable thermodynamically, respectively.

We also compute the optical responses of other monolayer TMDs with small energy difference and transition barrier, as shown in **Figure** **6**. One could see that in both MoSe_2_ and WSe_2_, the $\chi_{yy}^{\prime }\left(1T^{\prime },\omega \right)$ is higher than the other two in most frequency regimes. Thus, a *y*‐LTPL is able to drive a 2H→1T′ phase transition in these systems. Specifically, if the *y*‐LTPL frequency is selected to be 4.4 THz (for MoSe_2_) and 4.5 THz (for WSe_2_), intensity of 1.3 × 10^11^ (MoSe_2_) and 9.2 × 10^10^ W⋅cm^−2^ (WSe_2_) would be enough for the transition, respectively. If the frequency is low (below the IR‐active mode, e.g., 2 THz), then the required intensity would be higher, which is 1.2 × 10^12^ W⋅cm^−2^ for MoSe_2_ and 2.8 × 10^11^ W⋅cm^−2^ for WSe_2_. On the other hand, if one wants to facilitate phase transition from 1T′ to 2H, the frequency has to be chosen carefully (4.6 THz for both MoSe_2_ and WSe_2_). As for the monolayer WTe_2_, since its ground state is 1T′, we can apply a *y*‐LPTL with *ω* = 3.4 THz and *I* = 6.4 × 10^10^ W⋅cm^−2^ to drive a transition from 1T′ to 2H. Comparing Figures 5a and 6, the monolayer MoTe_2_ might be the best platform to realize ultrafast topological phase transition between the 2H and 1T′ phases back and forth, because it allows wider frequency windows that have both large $\chi_{yy}^{\prime }\left(2H,\omega \right)-\chi_{yy}^{\prime }\left(1T^{\prime },\omega \right)$ and $\chi_{yy}^{\prime }\left(1T^{\prime },\omega \right)-\chi_{yy}^{\prime }\left(2H,\omega \right)$.

### 3.5 Discussion

One has to note that defects and disorders always exist in real samples. If the defects arise in the material with a considerably high concentration, they may strongly affect the optical response functions, by significantly reducing the carrier lifetime and introducing localized doping levels in the phonon and electron band structures.^[^ ^31^ ^]^ For example, if the electronic doping level is shallow, it will enhance the THz optical absorption, and the unwanted optical loss would occur. In this case, the imaginary part of optical susceptibility needs to be considered. Actually, the optical absorption could also change the energy landscape and trigger phase transition.^[^ ^10^ ^]^ When the defect or impurity concentration is not high enough, we can phenomenologically incorporate its effect by adopting finite lifetime in the response formulae, Equations (5) and (6). The current theoretical work is based on the perfect crystal, and a complete evaluation on defects and impurities is out of scope here. We will discuss phase transitions in defective systems elsewhere.

There are several 3D bulk materials possessing different geometric phases with contrast topology. For example, bulk SnSe could have topologically trivial semiconducting phase *Pnma* and nontrivial phase $Fm\bar{3}m$ under ambient environment, and the phase transformation between them is also martensitic.^[^ ^1b^ ^]^ The present thermodynamic theory is applicable to these systems, as long as their spontaneous polarization *P*_s_ is zero. If the system has finite *P*_s_, additional terms need to be considered, as the THz oscillating electric field may flip *P*_s_ over time. As for the dimensionality effects, the free‐standing 2D materials are sandwiched by vacuum in the out‐of‐plane *z* direction, while the conventional 3D materials usually lack such free space. One has to note that during phase transformation, the associated transformation strain can be a few percent. For 2D materials, the transformation stress can be easily released by *z*‐motion, if they are freely suspended with slight pre‐buckling, so that long‐range elastic energy penalty can be minimal. In contrast, the accommodation of martensitic transformation strain in 3D bulk materials is a well‐known materials science problem,^[^ ^32^ ^]^ that causes long‐range elastic interactions and generally requires several orientation variants to cooperate,^[^ ^33^ ^]^ which constrains the speed and versatility. The residual back‐stress would also limit the spatial extent of the transformation and reduces the transformation reversibility. Hence, martensitic transitions in 2D materials can be greatly advantageous compared to 3D materials in speed and reversibility.

## 4 Conclusion

In summary, our theory on THz driven topological phase transition provides another route for optomechanical manipulations of the materials phase, by incorporating both photon–electron and photon–phonon coupling. In particular, this mechanism can be a novel way to realizing and understanding the topological phase transitions in monolayer TMDs under THz illumination, which can be potentially used in next‐generation data storage and computing devices based on 2D materials. In addition, such an optical scheme can be widely applicable to other materials for ultrafast martensitic transformations.

## 5 Experimental Section

##### Density Functional Theory

First‐principles calculations were based on DFT with spin–orbit coupling included self‐consistently, as implemented in the Vienna ab initio simulation package.^[^ ^34^ ^]^ The solid‐state Perdew–Burke–Enzerhof^[^ ^35^ ^]^ functional was adopted to evaluate exchange–correlation potential. Projector‐augmented wave method^[^ ^36^ ^]^ and planewave basis set were used to treat the core and valence electrons, respectively. Planewave cutoff energy was set as 350 eV. In order to eliminate the artificial image interactions under 3D periodic boundary condition, a vacuum space of 18 Å was adopted in the *z*‐direction. Monkhorst–Pack *k*‐mesh^[^ ^37^ ^]^ of (15 × 15 × 1) and (9 × 15 × 1) grids were used to optimize the unit cell geometry of 2H and 1T′ phase, and meshes of (45 × 45 × 1) and (25 × 45 × 1) were used to calculate the electron contributed frequency‐dependent susceptibility ${\overset{↔}{\chi }}^{\mathrm{el}}\left(\omega \right)$. Total energy and force convergence criteria were set to be 1 × 10^−7^ eV and 0.001 eV Å^−1^, respectively. The convergence of these parameters were well tested. Phonon dispersion and zone center phonon mode lifetime calculations were performed using density functional perturbation theory (DFPT)^[^ ^38^ ^]^ as implemented in the Phonopy^[^ ^39^ ^]^ and Phono3py^[^ ^40^ ^]^ codes. LO‐TO splitting effect was included, and the LO frequency near the Γ‐point was used to evaluate optical responses. Note that finite displacement method yielded similar results as in DFPT.

## Conflict of Interest

The authors declare no conflict of interest.

## Author Contribution

J.Z. conceived the project. J.Z., H.X., and Y.S. performed the calculations. J.Z., H.X., and J.L. derived the theory, analyzed data, and wrote the manuscript.

## Supporting information

Supporting Information

## Data Availability

The data that support the findings of this study are available from the corresponding author upon reasonable request.
