# Supplementary material for: Terahertz Driven Reversible Topological Phase Transition of Monolayer Transition Metal Dichalcogenides
Source: Adv Sci (Weinh). 2021 Apr 2;8(12):2003832. doi: 10.1002/advs.202003832 (PMC8224436; doi:10.1002/advs.202003832)
Supplement: Supplementary file 1 — Supporting Information [file ADVS-8-2003832-s001.pdf]

© 2021 Wiley-VCH GmbH

ADVANCED SCIENCE
Open Access

Supporting Information

for *Adv. Sci.,* DOI: 10.1002/advs.202003832

Terahertz Driven Ultrafast Reversible Topological Phase Transition of Monolayer Transition Metal Dichalcogenides

Jian Zhou[1,], Haowei Xu[2], Yongliang Shi[3], and Ju Li[2,4,†]*

Supporting Information for

Terahertz Driven Reversible Topological Phase Transition of Monolayer Transition Metal Dichalcogenides

Jian Zhou[1,*], Haowei Xu[2], Yongliang Shi[3], and Ju Li[2,4,†]

[1] Center for Alloy Innovation and Design, Center for Advancing Materials Performance from the Nanoscale, State Key Laboratory for Mechanical Behavior of Materials, Xi'an Jiaotong University, Xi'an 710049, China

[2] Department of Nuclear Science and Engineering, Massachusetts Institute of Technology, Cambridge, MA 02139, USA

[3] Center for Spintronics and Quantum Systems, State Key Laboratory for Mechanical Behavior of Materials, Xi'an Jiaotong University, Xi'an 710049, China

[4] Department of Materials Science and Engineering, Massachusetts Institute of Technology, Cambridge, MA 02139, USA

Emails: [*]J.Z. jianzhou@xjtu.edu.cn; [†]J.L. liju@mit.edu

Contents

1. More discussions on photon-phonon interaction in THz

2. Electronic band dispersion of monolayer $MoTe_2$

3. Three phonon scattering

4. LO-TO splitting effects

5. Ab initio molecular dynamics simulation

6. Transition saddle point properties

1. More discussions on photon-phonon interaction in THz

The phonon contributed susceptibility (Equation 6 in the main text) contains multiplications of two Born effective charge $Z^*_{\mu,ik}$, which is a second order interaction under electric field E on the total Gibbs free energy. One may ask if there is a lower order of interaction that would affect the interaction, which is proportional to $V\vec{E}\cdot\vec{P}_{\mathrm{in}}$. Here the $\vec{P}_{\mathrm{in}}$ indicates intrinsic phonon vibration (e.g. under thermal excitation) induced polarization (with phonon frequency ω_m) and static polarization (zero frequency), not the electric field induced polarizations (which is proportional to $\vec{E}$ and is evaluated through χ^{ph}). Actually, if the alternating electric field vector component is $\mathcal{E}_i(\omega,t)=E_i e^{\mathrm{i}\omega t}$. The intrinsic polarization component that is contributed from phonon vibrations is $P_{\mathrm{in},i}(\omega,t)=P_{\mathrm{s},i}+\sum_{\mu,k,m}Z^*_{\mu,ik}\tau^m_{\mu,k}e^{\mathrm{i}\omega_m t}$, where $P_{\mathrm{s},i}$ is possible static polarization (e.g. in ferroelectrics, with frequency $\omega_{\mathrm{s}}=0$), $Z^*_{\mu,ik}$ is Born effective charge component (i,k) on ion-μ, and τ is displacement of the mode-m. Their first order interaction is then

$$\sum_i \overline{\mathcal{E}_\iota(t)P_{\mathrm{in},\iota}(t)^*}=\sum_i \overline{E_\iota e^{\mathrm{i}\omega t}\sum_{\mu,k,m}Z_{\mu,\iota k}\tau^m_{\mu,k}e^{-\mathrm{i}\omega_m t}}=\sum_{i,\mu,k,m}E_i Z_{\mu,ik}\tau^m_{\mu,k}\,\overline{e^{\mathrm{i}(\omega-\omega_m)t}},$$

where $\bar{A}$ indicates time average of quantity A, and the static polarization $P_{\mathrm{s},i}$ contribution vanishes under time average. Clearly, this interaction only contributes when the light frequency ω overlaps the phonon (IR-active) frequency ω_m, which is proportional to $\delta(\omega-\omega_m)$, or $\mathrm{Im}\frac{1}{\omega_m-\omega-\mathrm{i}/\tau_m}$. Since in our theory, we do not consider such case (we need to avoid the frequency interval of $\left[\omega_m-\frac{1}{\tau_m},\omega_m+\frac{1}{\tau_m}\right]$ where IR-phonon absorption would occur, as indicated in the main text), this interaction can be omitted.

Note that the external electric fields can also lead to ion vibrations with photon frequencies ω, similar as in driven oscillators. These vibrations contribute to a polarization that is linearly dependent on the electric field, $P^{\mathrm{E}}_i(\omega,t)=\chi^{\mathrm{ph}}_{ii}(\omega)E_i(\omega)e^{\mathrm{i}\omega t}$, where χ^{ph} is the ion susceptibility. The interaction $P^{\mathrm{E}}_i(\omega,t)E_i$ is

a second order effect on the interaction and has two frequency components. The first one is $\chi_{ii}^{\mathrm{ph}}(\omega)E_i^2$, which is time independent, and contribute to the total Gibbs free energy, as we focus in the main text. The other is $\chi_{ii}^{\mathrm{ph}}(\omega)E_i^2e^{\mathrm{i}2\omega t}$, which has frequency 2ω. From energy point of view, the time average of this term is also zero and can be neglected. But from dynamics point of view, it does leads to an ion vibration at frequency 2ω that may influence the phase transition process. But the amplitude of such vibration is usually negligible.

2. Electronic band dispersion of monolayer $MoTe_2$

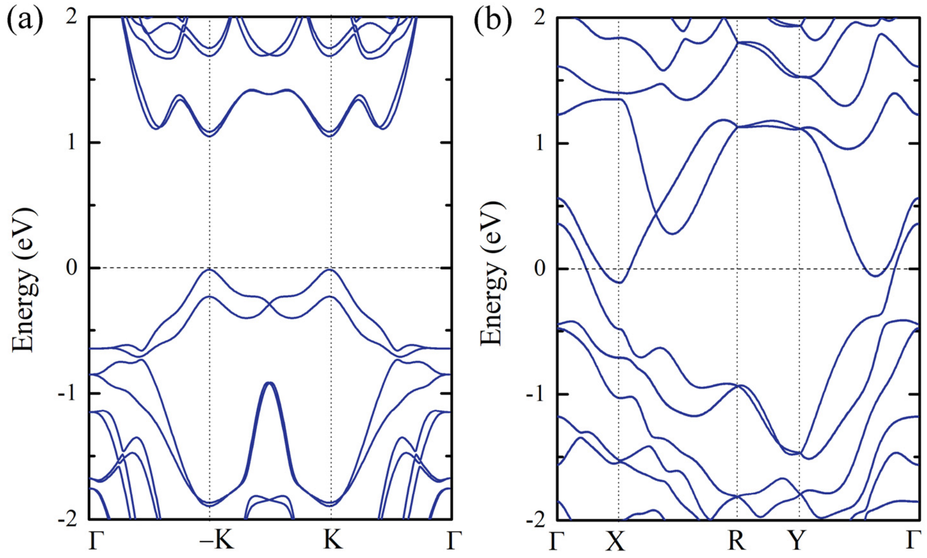

Figure S1. Electronic band structure of monolayer (a) 2H-$MoTe_2$ and (b) 1T′-$MoTe_2$.

3. Three phonon scattering

The interatomic potential $V(\{r_i\})$ can be expressed as

$$V(\{r_i\}) = V(\{r_i^0\}) + \sum_i \frac{\partial V}{\partial r_i}\delta r_i + \sum_{ij}\frac{1}{2!}\frac{\partial^2 V}{\partial r_i \partial r_j}\delta r_i \delta r_j + \sum_{ijk}\frac{1}{3!}\frac{\partial^3 V}{\partial r_i \partial r_j \partial r_k}\delta r_i \delta r_j \delta r_k$$

$$+ \sum_{ijkl}\frac{1}{4!}\frac{\partial^4 V}{\partial r_i \partial r_j \partial r_k \partial r_l}\delta r_i \delta r_j \delta r_k \delta r_l + \cdots$$

Here $i, j, k, l = 1, 2, \dots, 3N$, where N is the total number of atoms. $\{r_i^0\}$ are the equilibrium atomic positions, and $\delta r_i = r_i - r_i^0$ are the displacements (vibrations) around the equilibrium positions. The equation above is nothing but a standard Taylor expansion of $V(\{r_i\})$ around $\{r_i^0\}$. Note that $\{r_i^0\}$ are equilibrium positions, thus the first order terms $\frac{\partial V}{\partial r_i}$ vanish.

As is well-known, the phonon spectrum comes from the second order (harmonic) term $V^{(2)} = \frac{1}{2!}\frac{\partial^2 V}{\partial r_i \partial r_j}\delta r_i \delta r_j$. The phonons can be considered as a Fourier transformation of the atomic displacements ($\delta r_i = \sum_{q,n} u_{q,n} e^{\mathrm{i} q\cdot r_i}$, $u_{q,n}$ is the phonon mode, q and n are phonon wavevector and branch index, respectively), followed by a second quantization. Since the crystals are periodic in space, after the Fourier transformation, only terms involving one phonon modes ($u_{q,n}^* u_{q,n}$) can survive, while terms involving different phonons $u_{q,n}^* u_{q',n'}$ are unanimously zero. Therefore, $V^{(2)}$ describes a non-interacting phonon system, which corresponding to infinitely long phonon lifetime in perfect crystals.

However, the anharmonic terms would lead to interactions between the phonons. Putting the same Fourier transformation $\delta r_i = \sum_{q,n} u_{q,n} e^{\mathrm{i} q\cdot r_i}$ into $V^{(3)} = \frac{1}{3!}\frac{\partial^3 V}{\partial r_i \partial r_j \partial r_k}\delta r_i \delta r_j \delta r_k$ and $V^{(4)} = \frac{1}{4!}\frac{\partial^4 V}{\partial r_i \partial r_j \partial r_k \partial r_l}\delta r_i \delta r_j \delta r_k \delta r_l$, it can be seen that the $V^{(3)}$ and $V^{(4)}$ describe the three and four phonon interactions, respectively. These interactions lead to phonon scatterings and shorten the phonon lifetimes. Given a phonon scattering rate of Γ, then the phonon lifetime should be $\tau \sim 1/\Gamma$. Generally speaking, if the phonon displacements δr_i are not too large, then the third-order anharmonic term $V^{(3)}$ is much more significant than the fourth-order anharmonic

terms $V^{(4)}$, as in a typical Taylor expansion.[1] Hence, the phonon lifetimes are usually dominated by three phonon scattering. Also, the three-phonon scattering time (inverse phonon lifetime) scale approximately linearly with temperature, while the four-phonon scattering time scales approximately quadratically with temperature.[2] As a result, except for some particular materials (such as cubic BAs), the four-phonon scattering rate can be ignored, as long as the temperature is not too high. Indeed, there are other sources of phonon scattering, such as the scattering with defects, boundaries, etc. But these effects can be minimized with high quality samples.

4. LO-TO splitting effects

In ionic semiconductors, when a mode is both Raman and IR active, LO-TO splitting would occur near the zone center in the phonon dispersion. It could distinguish the mode that contributes to the dielectric permittivity constant along $\mathbf{q}$ ($\mathbf{q} \to 0$), which is longitudinal with higher frequency, and the mode that is perpendicular to the propagating direction, which is the transverse mode. In most cases, the eigenmode of mass averaged dynamic matrix $\tilde{C}(\mathbf{q} \to 0)$ is identical to $\tilde{C}(\mathbf{q} = 0)$, even though the frequency can be different.[3] In this case, the frequency can be evaluated by $\omega_m^2(\mathbf{q} \to 0) = \omega_m^2(\mathbf{q} = 0) + \frac{1}{V\epsilon_0}\frac{\sum_{\alpha\beta} q_\alpha S_{m,\alpha\beta} q_\beta}{\sum_{\alpha\beta} q_\alpha \epsilon_{\alpha\beta}^{\infty} q_\beta}$. According to symmetry group analysis, only the 2H phase phonon would have LO-TO splitting, which occurs on the E' mode. Our calculations indicate that the LO mode would increase its frequency by ~0.3 THz (Figure S2).

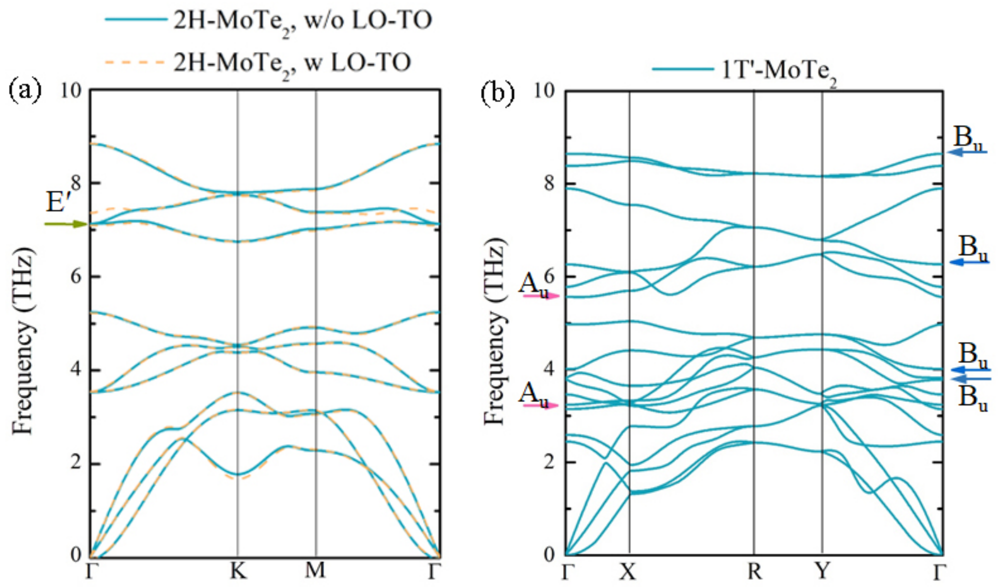

Figure S2. Phonon dispersion of (a) 2H-MoTe2 and (b) 1T′-MoTe2. The infrared-active modes are indicated by small arrows.

5. Ab initio molecular dynamics simulation

To confirm the prediction from thermodynamic theory (main text), we simulate effect of LPTL by applying time-dependent finite homogeneous electric field within the framework of Ab initio molecular dynamics. According to the Born-Oppenheimer approximation, the light electron moves fast enough to follow the motion of heavy nuclei, which moves in the timescale of femtosecond. The frequency of the applied alternating finite electric field in this report is in THz (picosecond timescale), which varies slowly enough to validate the Born-Oppenheimer approximation.

Electric enthalpy functional H of an insulator under an electric field can be written as,[4]

$$H = U_{\mathrm{KS}} - V_{\mathrm{f.u.}}\mathbf{P}\cdot\mathbf{E}$$

where U_{KS} is Kohn-Sham internal energy, $V_{\mathrm{f.u.}}$ is the unit cell volume. $\mathbf{P}$ is the screened macroscopic polarization, which is defined in the modern theory of polarization. This enthalpy implies that the total force acting on the ion-μ is $\mathbf{F}^{\mu}_{\mathrm{tot}} = \mathbf{F}^{\mu} + Z^{*}_{\mu}\mathbf{E}$, where $\mathbf{F}^{\mu}$ is the Hellmann-Feynman force calculated by Kohn-Sham equation and Z^{*}_{μ} is the Born effective-charge tensor on μ.[5] Both the Hellmann-Feynman force and the Born effective charge have been well-adopted in the VASP code. At any time t, we add an alternating electric field into the system according to $\mathbf{E}(t) = \mathbf{E}\sin\omega t$, where ω is the light frequency. In the current work, the direction of the electric field is along y (zigzag) of 2H-$MoTe_2$. A Verlet algorithm is used to integrate Newton's equation of motion over time, and we set the time step δt to be 1 fs. In the Verlet algorithm, the atom corrdinations $\mathbf{x}_{\mu}$ evolves according to finite difference method $\mathbf{x}_{\mu}(t+\delta t) = 2\mathbf{x}_{\mu}(t) - \mathbf{x}_{\mu}(t-\delta t) + \frac{\mathbf{F}^{\mu}_{\mathrm{tot}}(t)}{m_{\mu}}$, where m_{μ} is the atom mass.

During the simulation, the average displacement of each ions as a function of simulation time is plotted in Figure S3. One could see that the equilibrium structure is changed under alternating y-LPTL. In detail, Mo ions move to $+x$-position (0.007 Å), while Te ions move to $-x$-position (0.003 Å) from their intrinsic equilibrium positions. At the new equilibrium positions, they vibrate with frequency of 7.1 THz, which is the

Raman mode as discussed in the main text and confirmed experimentally.[6] In addition, small displacement along z is also detected. Note that such equilibrium structure change agrees with our thermodynamic analysis which is toward 2H→1T′ phase transition. Due to the limitation of modern theory of polarization, we could not directly apply large electric field onto small bandgap systems, such as transition saddle point and 1T′ phases, hence similar simulations are not performed on these states. Besides, one could see that double frequency effect (vibration with $2\omega = 2$ THz) is negligible, as discussed previously.

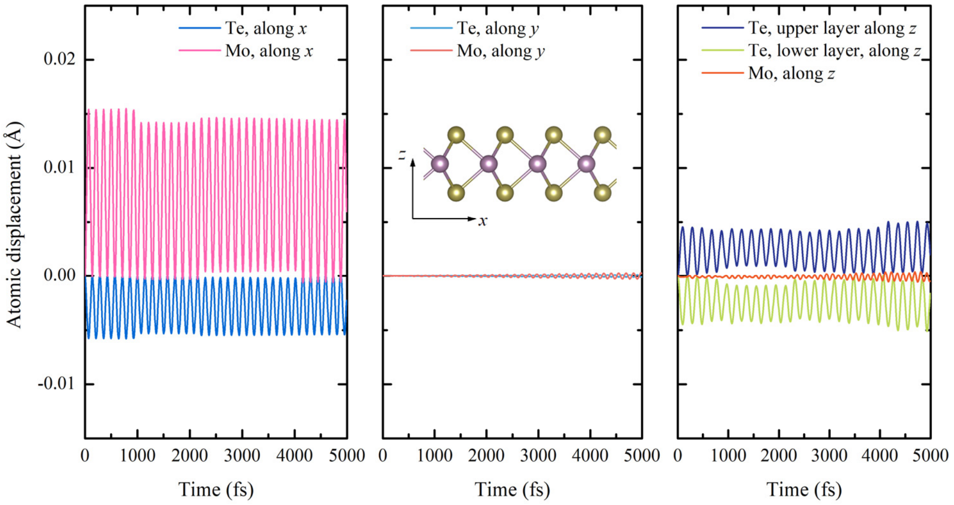

Figure S3. Atomic displacement variation of 2H-$MoTe_2$ under y-LPTL (frequency of 1 THz, magnitude of 1 V·nm^{-1}).

6. Transition saddle point properties

We perform nudged elastic band method to calculate the transition saddle point (SP) state along 2H→1T′ phase transition of $MoTe_2$. The energy profile, electronic and phonon dispersion, real part of total susceptibility, and the coordinates of SP are shown below (Figures S4-S6).

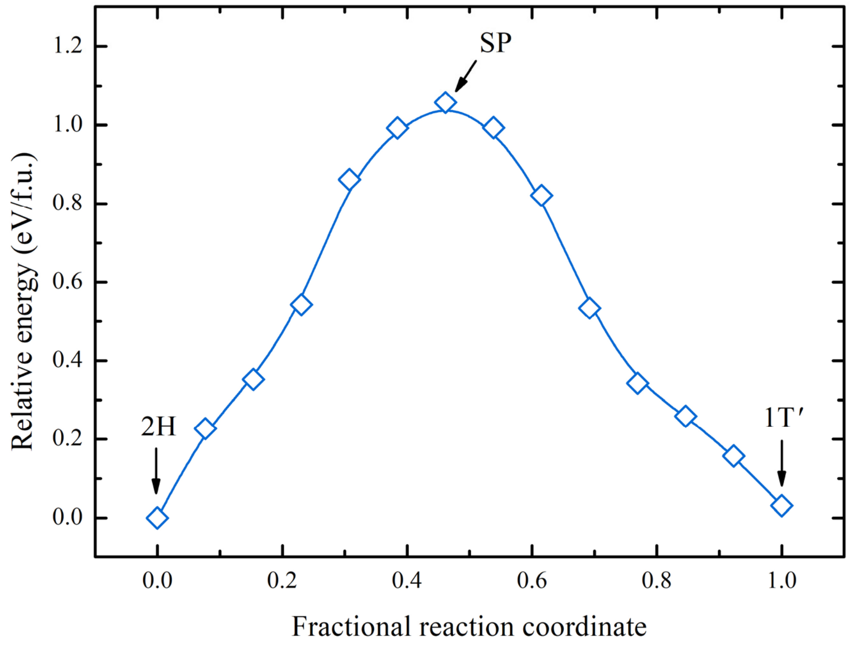

Figure S4. Relative energy as a function of reaction path between 2H and 1T′-$MoTe_2$ calculated by cell variable nudged elastic band method.

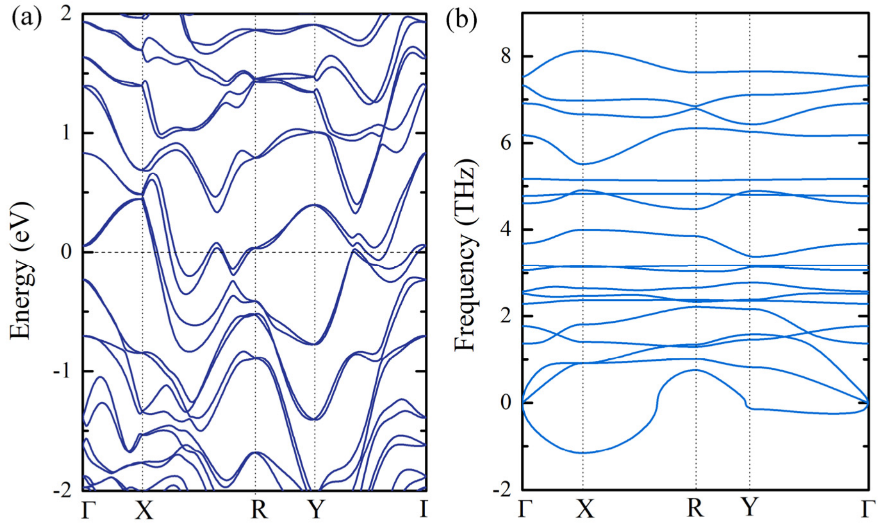

Figure S5. Electron and phonon dispersion of the transition saddle point state of $MoTe_2$.

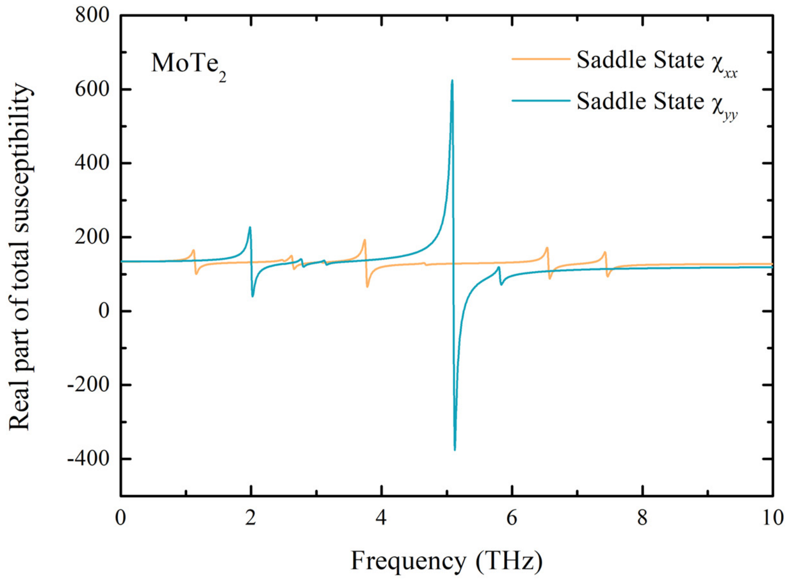

Figure S6. Frequency dependent susceptibility of the transition saddle point state of $MoTe_2$.

Transition saddle point state geometry (in VASP format)

```
SP structure MoTe2
   1.00000000000000
     6.2982786011128802     0.0000000000000000     0.0000000000000000
     0.0000000000000000     3.2949549763983565     0.0000000000000000
    -0.2001483637112157     0.0000000000000000    21.2350279439861467
   Te     Mo
     4       2
Direct
  0.1002385675198268  0.7499499342935820  0.6168156534193452
  0.6512468452444661  0.2499499342935820  0.5515952042847242
  0.3005647410147483  0.7499499342935820  0.4259929741380923
  0.7964223092761338  0.2499499342935820  0.3951300695108264
  0.9246611611376726  0.7499526033258022  0.4914277094152611
  0.2405726500015357  0.2499526033258022  0.5254867890399079
```

Reference:

[1] X. Yang, T. Feng, J. S. Kang, Y. Hu, J. Li, X. Ruan, *Phys. Rev. B* **2020**, 101, 161202.

[2] T. Feng, X. Ruan, *Phys. Rev. B* **2016**, 93, 045202.

[3] X. Gonze, C. Lee, *Phys. Rev. B* **1997**, 55, 10355.

[4] R. W. Nunes, X. Gonze, *Phys. Rev. B* **2001**, 63, 155107.

[5] H. X. Fu, L. Bellaiche, *Phys. Rev. Lett.* **2003**, 91, 057601.

[6] J. Shi, Y.-Q. Bie, W. Chen, S. Fang, J. Han, Z. Cao, T. Taniguchi, K. Watanabe, V. Bulović, E. Kaxiras, P. Jarillo-Herrero, K. A. Nelson, *arXiv* **2020**, 1910.13609.
